# Community-Based Survey Exploring Use of the Dietary Supplement Creatine by Adult Non-Athletes

**DOI:** 10.3390/nu13082529

**Published:** 2021-07-24

**Authors:** Melissa J. Benton, Jefferson M. Spicher, Sherry McCormick

**Affiliations:** Department of Nursing, Helen & Arthur E. Johnson Beth-El College of Nursing & Health Sciences, University of Colorado Colorado Springs, Colorado Springs, CO 80918, USA; jspiche2@uccs.edu (J.M.S.); smccorm3@uccs.edu (S.M.)

**Keywords:** creatine, dietitian, nutritionist, healthcare provider, fitness trainer, personal trainer, supplement use, nutrition education

## Abstract

Creatine is classified as a “sports supplement”, but it also has health benefits. The purpose of this study was to assess use of creatine as a dietary supplement in adult non-athletes. Three hundred ninety-nine adults (19–89 years) completed an online survey. Among the respondents, 77% (*n* = 307) were regularly active, including participation in weightlifting (34%), running (34%), and cycling (21%). Twenty-eight percent (*n* = 111) reported use of creatine with an average dose of 6.4 ± 4.5 g. Daily creatine use was reported by 45%, and 38% reported using creatine 2–6 times weekly. Primary sources of information about creatine were trainers/coaches (29%), friends/family (32%), and internet (28%). Forty percent (*n* = 44) of creatine users were female. When compared by age, 46% of young, 32% of midlife, and 6% of old respondents reported creatine use with no differences in dose or frequency. Young and midlife respondents reported primarily trainers/coaches, friends/family, and internet as sources of information about creatine, but old respondents limited their sources to friends/family and fitness magazines. Although creatine is widely used by adult non-athletes who regularly exercise, dietitians and other healthcare providers are not the primary source of information. Fitness trainers can appropriately provide guidance and education regarding safe and effective use of creatine.

## 1. Introduction

Use of the dietary supplement creatine is widely reported among athletes, including more than one-third of soccer players and more than two-thirds of football players [1]. However, there are differences based on gender, and males report more frequent use of creatine than females [1]. Gender differences have also been observed in the armed forces. Approximately 39% of male soldiers report using creatine compared with only 5% of female soldiers [2]. This difference has been attributed to a greater interest by males in the ergogenic effects of creatine that decrease muscle damage related to training and increase muscle mass, strength, and power [1,2,3,4,5,6,7]. These effects enhance performance during sports and high-intensity activities that are dependent on adenosine triphosphate (ATP) as a fuel source [4,8]. 

Creatine is naturally available in the diet. However, it is primarily found in meat and seafood [9,10], so individuals with limited consumption of animal products such as vegetarians or vegans may not consume adequate creatine without supplementation [9,10]. In addition, normal aging is associated with decreased meat consumption, so creatine supplementation may be necessary and has been recommended for older adults in order to maintain health and function [9,11,12]. 

As a dietary supplement, creatine is classified as a “sports supplement” [13]. However, there is a well-established body of evidence demonstrating the potential health benefits of creatine. In its metabolic role related to the synthesis of ATP, creatine improves mitochondrial efficiency and reduces formation of reactive oxygen species [12,14]. In addition to possible benefits related to sarcopenia and frailty with aging [15,16], creatine supplementation has been explored for management of ischemic heart disease [17], dyslipidemia [18], diabetes [19,20], cancer [21,22], osteoarthritis [23], fibromyalgia [24], and depression [25]. Furthermore, long-term observational and experimental studies lasting up to eight years have provided a substantial body of evidence that creatine supplementation with up to 10 g per day is safe and does not impair renal function [6,7,16,26]. 

In the last two decades, participation in muscle-strengthening exercise has increased among men and women in all age groups [27,28]. Approximately 20% of adults in the United States and Europe report regular training at least two days a week [29,30]. As the number of adults who regularly exercise grows, the need to understand their behaviors and lifestyle choices becomes increasingly important in order to provide appropriate guidance and education to ensure safe and effective exercise-related behaviors. 

Among adult non-athletes, use of creatine is not well understood. A 1998–1999 U.S. population-based survey of more than 10,000 adults found that 3.6% of male respondents reported use of creatine while female respondents did not [31]. Subsequently, the 2007 U.S. National Health Interview Survey found a 3% overall prevalence of creatine use, but gender-specific data were not reported [32]. By comparison, a 40% prevalence of creatine use was reported in a smaller study of 229 civilian and military health club members who were predominantly male [3]. In addition to these wide variations in prevalence, evidence regarding patterns of use and sources of information regarding creatine is limited. Therefore, the primary aim of this study was to assess the prevalence and patterns of use of creatine as a dietary supplement in adult non-athletes. Sources of information about creatine and current health conditions were assessed as a secondary aim.

## 2. Materials and Methods

### 2.1. Design

An online survey administered through Qualtrics (Qualtrics^XM^, Provo, UT, USA) was used to collect data. The survey included 11 questions about demographics, anthropometrics, physical activity patterns, use of creatine including dosage and frequency, sources of information regarding creatine, and current medical diagnoses. Two questions (age and creatine dosage) were open ended, and the remaining nine questions were closed choice, with respondents answering yes/no or selecting from a pre-determined list of responses. Respondents were recruited by email and Facebook using a snowball sampling strategy and were asked to complete the questionnaire. Data were collected between September 2020 and March 2021. All responses were anonymized and aggregated for analysis.

### 2.2. Participants

Inclusion criteria were a willingness to complete the questionnaire and self-reported age of 19 years or older. The study was approved by the University of Colorado Colorado Springs Institutional Review Board, and all participants indicated their willingness to voluntarily participate in the study prior to accessing the survey.

### 2.3. Survey

The study questionnaire included 11 items in 4 general categories: (1) demographic information; (2) anthropometric and physical activity data; (3) creatine use; and (4) health information. Items were based on previous questionnaires regarding use of creatine and other dietary supplements [3,33,34] and on recommendations for future research derived from a meta-analysis of supplement use by athletes [1]. To decrease respondent burden and facilitate completion, questions were structured for brief fill-in or check-the-box responses. All items were optional, and participants were able to skip any questions they did not wish to answer. 

To characterize physical activity, respondents were asked “On average, do you engage in moderate-vigorous physical activity for at least 30 min on two or more days of the week? Moderate-vigorous activity is at an intensity that slightly increases your heart rate or breathing and makes it somewhat difficult to carry on a conversation.” 

To characterize creatine use, respondents were asked a series of three questions, “Do you now use, or have you used in the past, the dietary supplement creatine?” “When you used creatine, how frequently did you use it?” and “When you used creatine, how much did you take in grams each day? One teaspoon is approximately equal to 5 g of creatine.” These were followed by a single question regarding sources of information about creatine, “How did you learn about creatine?” with a list of possible sources from which to choose that were identified from previous research [2,3,34]. Only respondents who answered yes to the use of creatine had the option of answering the questions regarding frequency, dose, and sources of information.

To obtain health information, respondents were asked a single question, “Has a physician, nurse practitioner, or other health professional ever told you that you had any of the following?” The phrasing of this question is consistent with the U.S. Behavioral Risk Factor Surveillance System questionnaire used to assess health diagnoses among U.S. residents [35]. The question was followed by a list of diagnoses for which creatine supplementation has been identified as beneficial by the International Society of Sports Nutrition [6]. 

### 2.4. Statistical Analysis

All data were analyzed using SPSS version 27 (IBM Corp., Armonk, NY, USA). Descriptive statistics were used to analyze participant characteristics and because data for creatine were not normally distributed, the Mann–Whitney U test was used to compare two groups based on use of creatine (yes/no). For subgroup analysis, data were grouped by age to allow comparison between young (19–35 years), midlife (36–65 years), and old (66–89 years) adults. The Kruskal–Wallis test was used for three-group comparison and when a statistically significant difference was identified, a Mann–Whitney U test was used for post hoc analysis [36]. Data are reported as frequencies (%) or means ± standard deviations, and significance was set at *p* < 0.05. 

## 3. Results

### 3.1. Sample Characteristics

A total of 399 questionnaires were completed online. Twelve respondents did not answer one or more demographic or anthropometric questions, but did answer the question regarding creatine use and so their questionnaires were retained for analysis. Data for all respondents are reported in Table 1. The respondents ranged in age from 19–89 years. The majority were women and reported their race as White. Overall, they were overweight based on self-reported height and weight, although body mass index (BMI) values ranged from underweight (16.3 kg/m^2^) to class III obesity (47.9 kg/m^2^). More than three-quarters of the respondents engaged in regular moderate-vigorous physical activity, with running, weightlifting, and cycling being the most popular, although 37% of respondents reported “other” activities that were not defined. The most frequently reported health diagnoses were high cholesterol and depression, followed by cancer, osteoarthritis, and high triglycerides.

### 3.2. Use of Creatine 

Twenty-eight percent (*n* = 111) of respondents reported current or past use of creatine. Of those, 96% (*n* = 107) also reported their frequency of use and 92% (*n* = 102) reported their dosage. The average dosage was 6.4 ± 4.5 g with the frequency most often reported as daily. The most common sources of information regarding creatine were friends/family, followed closely by trainers/coaches and the internet. However, an equal number of respondents also reported “other” sources of information that were not defined.

When respondents were compared by use of creatine (yes/no), there were significant between-group differences (Table 2). Those who reported current or previous use of creatine were younger and had a higher body mass index (BMI). They were also predominantly males compared with the non-creatine users who were predominantly female. However, it is notable that among creatine users, the gender gap was narrower, with a 60:40 ratio of males to females, compared with a 25:75 ratio among non-users. Not surprisingly, 90% of creatine users were physically active compared with only 72% of non-users. A significantly greater percentage of creatine users reported running, cycling, and weightlifting, with weightlifting and running being the two most frequently reported activities. Creatine users also reported a significantly lower frequency of high cholesterol, high triglycerides, osteoarthritis, depression, and cancer. 

### 3.3. Use of Creatine by Age Group

For analysis by age, respondents were grouped as young (*n* = 125), midlife (*n* = 137), and old (*n* = 126). There were no significant anthropometric or gender differences between age groups (Table 3). The young group was significantly more physically active than the midlife and old groups, with greater participation in running, weightlifting, and team/group sports. By comparison, the old and midlife groups reported significantly greater participation in other (undefined) activities. Respondents in the young group also reported significantly lower frequencies of all health diagnoses than either the midlife or old groups. The frequency of creatine use was also highest in the young group (46%), followed by the midlife group (32%), with only 6% of the old group reporting current or past use. However, among those who did report use of creatine in each age group, there were no statistically significant differences in dosage or frequency of use. Among young respondents, the internet was the most frequently identified source of information about creatine, followed by trainers/coaches, friends/family, or other (undefined) sources. Among midlife respondents, trainers/coaches, friends/family or other (undefined) sources were the most frequently reported. Interestingly, old respondents reported obtaining information regarding creatine only from friends/family, fitness magazines, and other (undefined) sources. 

## 4. Discussion

To the authors’ knowledge, this is the first community-based survey exploring the use of creatine among a diverse group of adult non-athletes. Although two previous studies have reported use of creatine in non-athletes, their populations were limited to civilian and military health club members [3] or military soldiers in training [2]. In those studies, the prevalence of creatine use ranged from 38% to 40%, which was notably higher than the 28% prevalence found in the current study. However, those studies were predominantly (77–96%) male, while our sample was predominantly (65%) female. Furthermore, when the creatine users in our study were broken down by gender, females made up 40%, which was remarkably different from the previous studies in which no female civilian and military health club members [3] and only 5% of female soldiers [2] reported use of creatine. These differences most likely reflect an increased awareness of the benefits of creatine and participation by women in regular strength training over the past decade [27].

The wide age distribution among the respondents in the current study is also noteworthy. Although it was not altogether unsurprising that younger respondents reported higher rates of creatine use, there were 6% of respondents between the ages of 66–89 years who also reported either current or past use of creatine. In addition, they reported an average daily dose of approximately 6 g, which was similar to the average dose reported by young and midlife respondents and consistent with the recommended dose of 5 g per day that has been found to improve upper and lower body strength and power [37,38]. There are more than 53 million adults between the ages of 66–89 years in the United States alone [39]. They represent a population that can potentially benefit from use of creatine, although their sources of information are limited. Only the respondents in this age group failed to identify trainers and coaches as a source of information regarding creatine. This may be due to lack of awareness on the part of trainers who work with adults in this age group but who may not perceive their potential interest in creatine supplementation either for performance or health reasons. Given that 72% of the old respondents in the current study reported regular physical activity that could bring them into contact with fitness trainers at health clubs, recreation centers, gyms, and exercise studios, there is an opportunity for trainers to provide valuable education regarding the potential benefits of creatine.

Our findings regarding health diagnoses further support the need for education regarding not only the effects on performance but also the health benefits of creatine. Not surprisingly, both midlife and old respondents reported more frequent health diagnoses for which creatine has potential benefits. They also reported significantly less use of creatine. Although education is clearly warranted, our results demonstrate that it is not being provided by dietitians, nutritionists, pharmacists, or healthcare providers. In fact, these providers were not identified by respondents of any age as primary sources of information regarding creatine. This is unfortunate and represents a potential gap in counseling. 

Fitness trainers can fill the gap by providing effective education in the physical activity setting. Currently, there are more than 350,000 fitness trainers working in health clubs, recreation centers, gyms, and exercise studios in the United States [40] who regularly provide health-related guidance and education to clients [41]. Although there is no standardized requirement for nutrition education among personal trainers [42], certifying organizations such as the National Strength and Conditioning Association and the American College of Sports Medicine recommend competency in the areas of lifestyle and health, chronic disease, and nutrition [43]. Based on these competencies, education regarding use of creatine falls within their scope of practice.

We recognize that there are limitations to the generalizability of our findings. Currently there are multiple forms of creatine available as a dietary supplement [44]. Among these, creatine monohydrate is the most well-recognized and researched [7,44]. In two recent meta-analyses of creatine use, creatine monohydrate was the form of creatine reported by more than 75% of the included studies [26,38]. Although there are generally no safety concerns regarding creatine supplementation, the vast majority of safety and efficacy studies have focused on the use of creatine monohydrate [7,44]. Furthermore, in the United States, Canada, and the European Union, creatine monohydrate is the only form of creatine with regulatory approval as a dietary supplement [7]. Although it may be considered a weakness in our study design that we did not ask survey respondents to identify the form of creatine they used, our design is consistent with previous research with athletes [45,46] and non-athletes [2,3], and we believe it allows our findings to be more readily compared with those studies.

Other limitations include the use of self-report, which can create a risk of recall bias. Although our survey questions were based on previous questionnaires regarding use of creatine and other dietary supplements [1,2,3,34] and national population-based health surveys [35], it is possible that respondents did not accurately report their use of creatine or their health and demographic characteristics. In addition, use of an online survey format may have excluded some participants with limited online access. However, at least 85% of households in the United States currently have internet access [47], so we do not believe this was a major weakness in our study design. Our use of snowball sampling rather than random sampling may also have been a limitation. Non-probability sampling, which includes snowball sampling, is a more restrictive strategy that can limit representation based on the participants who are initially identified and recruited [48]. However, when recruiting through emails and social media where random sampling may not be possible, snowball sampling may be the most effective method for increasing enrollment in order to capture a wider sample.

We also believe there are recognizable strengths to our study. The wide age range of our respondents reflects participation by a representative sample of adults, and our sample included a greater percentage of female respondents than previous studies. Future research is needed with larger and even more diverse samples regarding creatine use in the community and the effect of education among non-athletes, especially older adults who can accrue both performance and health benefits from creatine. 

## 5. Conclusions

Our findings demonstrate that approximately one-third of adult non-athletes report current or past use of creatine. However, the prevalence is lower among midlife and old respondents who are more likely to accrue health as well as performance benefits from creatine use. Given the numerous documented benefits of creatine [15,16,17,18,19,20,21,22,23,24,25], it is unfortunate that education is not being provided by dietitians and other healthcare providers. Especially in the older population, the ability of creatine to increase or at the very least preserve muscle mass can have significant benefits. With growing numbers of adults regularly engaging in exercise, fitness trainers can fill the knowledge gap regarding safe and effective use of creatine for both performance and health. 

## Figures and Tables

**Table 1:** Characteristics of all (*N* = 399) respondents.

		Number (%)	Mean ± SD
Age (years)	(*n* = 388)		50.1 ± 21.1
Height (cm)	(*n* = 395		170.0 ± 9.9
Weight (kg)	(*n* = 394)		75.0 ± 17.8
Body mass index (kg/m^2^)	(*n* = 391)		25.9 ± 5.4
Gender	(*n* = 396)		
Female		259 (65%)	
Male		135 (34%)	
Transgender		2 (0.5%)	
Race/Ethnicity ^a^			
Black		14 (4%)	
White		354 (89%)	
Hispanic		35 (9%)	
Asian		10 (3%)	
Other		4 (1%)	
Physical Activity Participation			
Yes		307 (77%)	
No		92 (23%)	
Physical Activities ^b^	(*n* = 307)		
Running		135 (34%)	
Cycling		85 (21%)	
Swimming		15 (4%)	
Weightlifting		134 (34%)	
Team/Group		37 (9%)	
Other		147 (37%)	
Health Diagnoses			
Heart Disease		32 (8%)	
High Cholesterol		114 (29%)	
High Triglycerides		45 (11%)	
Diabetes		25 (6%)	
Osteoarthritis		45 (11%)	
Fibromyalgia		15 (4%)	
Depression		88 (22%)	
Cancer		47 (12%)	
Creatine Use			
Yes		111 (28%)	
No		288 (72%)	
Creatine Frequency	(*n* = 107)		
Daily		50 (45%)	
2–6 times per week		42 (38%)	
Once a week		3 (3%)	
Less than once a week		12 (11%)	
Creatine Dose (g)	(*n* = 102)		6.4 ± 4.5
Sources of Information			
Trainer/Coach		32 (29%)	
Friend/Family		35 (32%)	
Fitness Magazine		18 (16%)	
TV/Radio		3 (3%)	
Internet		31 (28%)	
Dietitian/Nutritionist		12 (11%)	
Nurse Practitioner		1 (1%)	
Physician		2 (2%)	
Pharmacist		0	
Other		34 (31%)	

Data are based on *N* = 399 unless otherwise indicated. **^a^** Total responses exceed 399 because some respondents indicated more than one race/ethnicity. **^b^** Total responses exceed 307 because some respondents indicated more than one activity.

**Table 2:** Between-group comparison of respondents based on use of creatine.

	Creatine	No Creatine	*p*-Value
	(*n* = 111)	(*n* = 288)	
Age (years)	37.0 ± 14.8	55.2 ± 21.0	**<0.001**
Weight (kg)	79.6 ± 16.8	73.3 ± 17.9	**<0.001**
Body mass index (kg/m^2^)	26.4 ±5.1	25.7 ± 5.5	**<0.001**
Gender			**<0.001**
Female	44 (40%)	215 (75%)	
Male	65 (59%)	70 (24%)	
Transgender	1 (1%)	1 (1%)	
Physical Activity Participation			**<0.001**
Yes	100 (90%)	207 (72%)	
No	11 (10%)	81 (28%)	
Physical Activities			
Running	52 (47%)	83 (29%)	**0.001**
Cycling	34 (31%)	51 (18%)	**0.005**
Swimming	6 (5%)	9 (3%)	0.284
Weightlifting	74 (67%)	60 (21%)	**<0.001**
Team/Group	9 (8%)	28 (10%)	0.619
Other	36 (32%)	111 (39%)	0.258
Health Diagnoses			
Heart Disease	6 (5%)	26 (9%)	0.233
High Cholesterol	12 (11%)	102 (35%)	**<0.001**
High Triglycerides	6 (5%)	39 (14%)	**0.021**
Diabetes	6 (5%)	19 (7%)	0.66
Osteoarthritis	2 (2%)	43 (15%)	**<0.001**
Fibromyalgia	3 (3%)	12 (4%)	0.491
Depression	17 (15%)	71 (25%)	**0.044**
Cancer	5 (5%)	42 (15%)	**0.005**

Data reported as mean ± standard deviation or frequency (%). *p*-values meeting the predetermined level of significance are bolded.

**Table 3:** Between-group comparison of respondents based on age.

	Young	Midlife	Old
	(*n* = 125)	(*n* = 137)	(*n* = 126)
Age (years)	25.7 ± 5.0	50.2 ± 9.3 *	75.1 ± 5.5 *^†^
Body mass index (kg/m^2^)	25.0 ± 5.0	26.5 ± 6.0	25.9 ± 4.9
Gender			
Female	72 (58%)	94 (69%)	87 (70%)
Male	52 (41%)	43 (31%)	37 (30%)
Transgender	2 (2%)	0	0
Physical Activity Participation			
Yes	109 (86%)	102 (75%) *	89 (72%) *
No	18 (14%)	35 (25%) *	35 (28%) *
Physical Activities			
Running	63 (50%)	52 (38%)	16 (13%) *^†^
Cycling	28 (22%)	34 (25%)	21 (17%)
Swimming	3 (2%)	6 (4%)	5 (4%)
Weightlifting	67 (53%)	49 (36%) *	16 (13%) *^†^
Team/Group	20 (16%)	7 (5%) *	9 (7%) *
Other	32 (25%)	51 (37%) *	61 (49%) *
Health Diagnoses			
Heart Disease	2 (2%)	4 (3%)	24 (20%) *^†^
High Cholesterol	10 (8%)	26 (19%) *	72 (58%) *^†^
High Triglycerides	5 (4%)	10 (7%)	29 (23%) *^†^
Diabetes	1 (1%)	9 (7%) *	14 (11%) *
Osteoarthritis	1 (1%)	8 (6%) *	35 (28%) *^†^
Fibromyalgia	0	4 (3%)	11 (9%) *^†^
Depression	18 (14%)	31 (23%)	37 (30%) *
Cancer	3 (2%)	13 (10%) *	28 (23%) *^†^
Creatine Use			
Yes	58 (46%)	44 (32%) *	7 (6%) *^†^
No	69 (54%)	93 (68%) *	117 (94%) *^†^
Creatine Frequency			
Daily	25 (20%)	20 (15%)	5 (4%)
2–6 times per week	24 (19%)	15 (11%)	1 (1%)
Once a week	2 (2%)	1 (1%)	0
Less than once a week	5 (4%)	6 (4%)	1 (1%)
Creatine Dose (gm)	5.8 ± 3.2	7.1 ± 5.9	6.7 ± 4.1
Sources of Information			
Trainer/Coach	18 (14%)	14 (10%)	0 *^†^
Friend/Family	17 (13%)	15 (11%)	2 (2%) *^†^
Fitness Magazine	4 (3%)	11 (8%)	3 (2%)
TV/Radio	1 (1%)	2 (2%)	0
Internet	23 (18%)	8 (6%)*	0 *^†^
Dietitian/Nutritionist	8 (6%)	4 (3%)	0 *
Nurse Practitioner	0	1 (1%)	0
Physician	0	2 (2%)	0
Pharmacist	0	0	0
Other	17 (13%)	13 (10%)	3 (2%)

Data reported as mean ± standard deviation or *n* (%). Young = 19–35 years; Midlife = 36–65 years; Old = 66–89 years. * Significantly different than Young (*p* < 0.05). ^†^ Significantly different than Midlife (*p* < 0.05).

## Data Availability

The data presented in this study are available on request from the corresponding author.
